# Trichite features contribute to the revision of the genus *Strombidium* (Alveolata, Ciliophora, Spirotricha)

**DOI:** 10.1111/jeu.13001

**Published:** 2023-09-25

**Authors:** Sabine Agatha, Birgit Weißenbacher, Michael Kirschner, Maximilian H. Ganser

**Affiliations:** ^1^ Department of Environment and Biodiversity Paris Lodron University of Salzburg Salzburg Austria; ^2^ School of Life Sciences Technical University of Munich Munich Germany

**Keywords:** marine plankton, Oligotrichida, systematics, taxonomy, ultrastructure

## Abstract

*Strombidium* is a species‐rich genus of oligotrichid ciliates mainly inhabiting the marine pelagial. In molecular phylogenies, the genus emerged as non‐monophyletic, and cladistic analyses suggest that it is largely characterized by plesiomorphies. A reliable split of the genus and the establishment of new genera necessitate, however, support by novel morphological and/or ultrastructural features. In the present study, the arrangement and ultrastructure of trichites are proposed as taxonomically relevant characters. *Strombidium biarmatum* Agatha et al., 2005 differs in the trichite pattern from the type species *Strombidium sulcatum* and most congeners. Aside from the trichites inserting anteriorly to the girdle kinety and generating the typical funnel‐shaped complex in the posterior cell portion, the species displays additional trichites between the adoral membranelles even visible in live cells. Here, this exceptional trichite arrangement is detailed based on transmission electron microscopic investigations. In molecular phylogenies, *S. biarmatum* forms a monophylum with two congeners sharing its trichite arrangement. Therefore, the strombidiid genus *Heteropilum* nov. gen. is established with *S. biarmatum* as type species to also include *H. paracapitatum* (Song et al., 2015) nov. comb. and *H. basimorphum* (Martin & Montagnes, 1993) nov. comb. Further differences discovered in the trichite ultrastructure support the organelles' taxonomic significance.

## INTRODUCTION

*Strombidium* Claparède & Lachmann, 1859 is a speciose genus of oligotrichid ciliates mainly inhabiting the marine pelagial. Its species are characterized by a C‐shaped adoral zone of membranelles at their apical cell ends and usually two dikinetidal somatic kineties, viz., a horizontally orientated, circular girdle kinety and a longitudinal ventral kinety. Trichites (oligotrichid extrusomes) insert anteriorly to the girdle kinety, forming a funnel‐shaped complex in the posterior cell portion covered by the polysaccharide platelets of the hemitheca. According to cladistic analyses, the only apomorphies that the genus *Strombidium* obtained during oligotrichid evolution were the horizontal arrangement of the girdle kinety and the longitudinal orientation of the ventral kinety; however, these features are synapomorphies also present in the ancestors of the families Cyrtostrombidiidae, Pelagostrombidiidae, and Tontoniidae as well as in some strombidiid genera (*Foissneridium*, *Opisthostrombidium*, *Williophrya*). Thus, *Strombidium* is largely characterized by plesiomorphies compared to the abovementioned families and genera, which later developed distinct innovations (Agatha, 2004; Agatha & Strüder‐Kypke, 2014). In molecular phylogenies, *Strombidium* is non‐monophyletic (Song et al., 2021); yet, a reliable split and the establishment of new genera need to be supported by novel morphological and/or ultrastructural features (Agatha, 2004). The position of the trichite attachment sites was already used for the establishment of the strombidiid genera *Foissneridium* and *Opisthostrombidium* (Agatha, 2011), for example.

Trichites were first reported as “Trichocysten” by Bütschli (1873) and subsequently often described from live and preserved cells. They were occasionally considered skeletal elements (Fauré‐Fremiet, 1924; Fauré‐Fremiet & Ganier, 1970) or contractile fibers (Lohmann, 1908), although Entz Sr. (1884), Penard (1920), and Kahl (1932) noticed an ejection of these organelles. Since then, the ejection of trichites by oligotrichids sampled from marine and freshwater has been repeatedly verified in several studies, using live observation and electron microscopy (Agatha, 2003; Agatha & Riedel‐Lorjé, 1997; Agatha et al., 2005; Bardele et al., 2018; Krainer, 1991; Modeo et al., 2001; Montagnes et al., 1988; Petz & Foissner, 1992; Rosati & Modeo, 2003). The organelles fulfill the definition of extrusomes given by Hausmann (1978), being rapidly dischargeable, membrane‐bound structures that undergo morphological changes during their transition from the resting state to the ejected form. Still, their function is unclear in oligotrichids; an involvement in defense or food capture is discussed.

In the type species *Strombidium sulcatum* Claparède & Lachmann, 1859, the trichites insert in a horizontal stripe directly anteriorly to the girdle kinety and extend obliquely into the cytoplasm, forming a funnel‐shaped complex in the posterior cell portion (Claparède & Lachmann, 1859; Fauré‐Fremiet, 1911, 1912; Fauré‐Fremiet & Ganier, 1970; Granda & Montagnes, 2003). Additional trichites associated with the adoral membranelles were first found in the freshwater species *Strombidium rehwaldi* (Petz & Foissner, 1992). In the following years, further congeners had been described sharing this feature, viz., *S. biarmatum*, *S. basimorphum* (Liu et al., 2011), and *S. paracapitatum* (Song et al., 2015). Representative 18S rRNA gene sequences of the latter three species form a statistically fully supported clade (99% Bayesian Inference probabilities, 100% Maximum Likelihood Bootstrap) in recent phylogenies (Li et al., 2020; Santoferrara et al., 2017; Song et al., 2015, 2019, 2021).

In the present study, we confirm the trichite arrangement in *Strombidium biarmatum* Agatha et al., 2005 by transmission electron microscopy, providing the first ultrastructural data on its two types of trichites. Based on morphologic and molecular data, a new genus is introduced with *S. biarmatum* as type species. The comparison of the trichites and their associated structures among oligotrichids reveals promising features for a further revision of the genus *Strombidium*.

## MATERIALS AND METHODS

### Sampling

**FIGURE 1:**
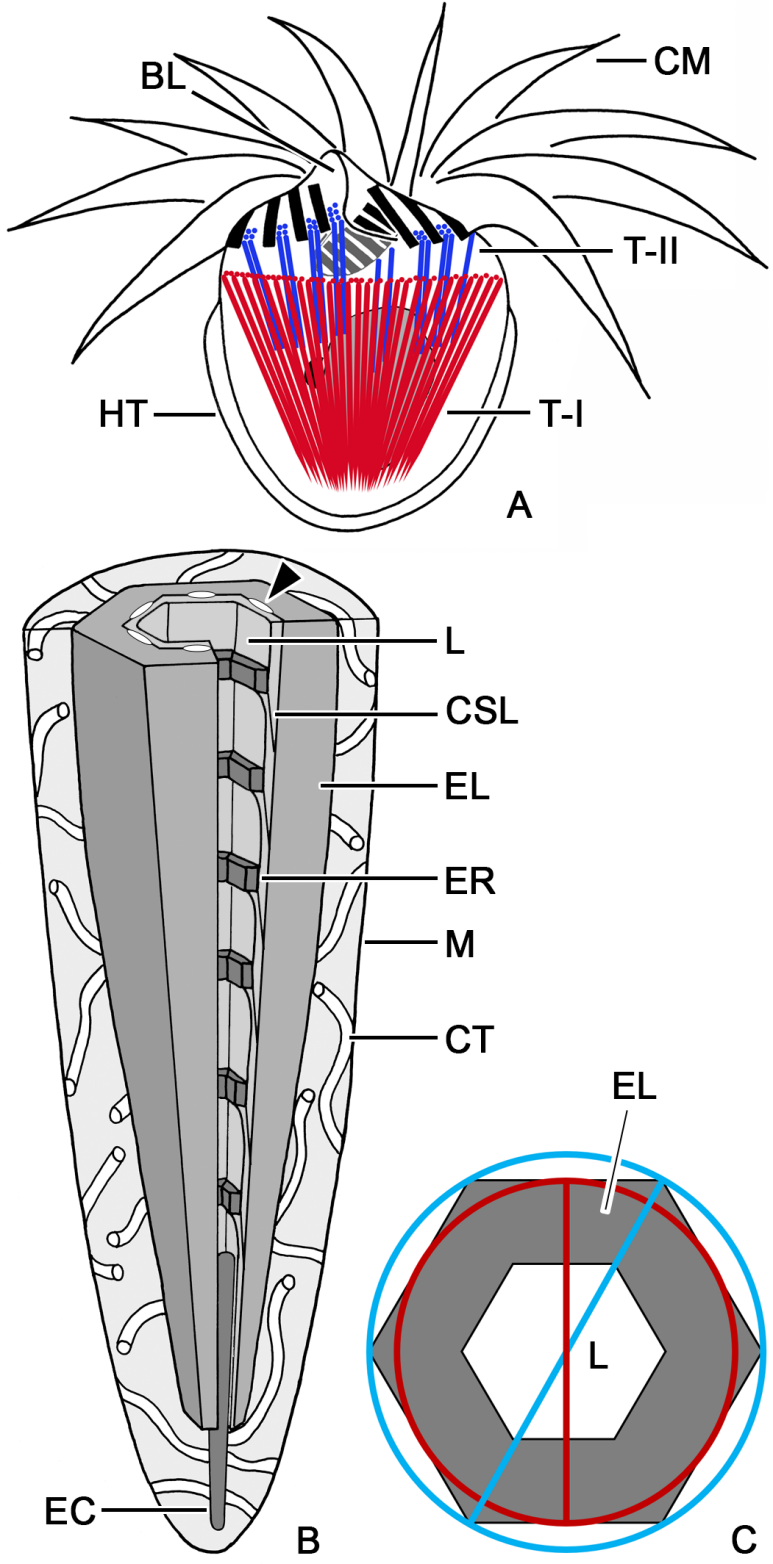
Trichites in *Strombidium biarmatum*. (A) Scheme showing the trichites (red; Type I) forming the common funnel‐shaped complex in the posterior cell portion and additional trichites (blue; Type II) inserting between the large collar and small buccal membranelles and in the buccal lip. (B) Cutaway drawing of a Type I trichite. An electron‐dense core fills the proximal portion of the lumen. Arrowhead denotes lacunas. (C) Scheme illustrating the incircle (red) and circumcircle (blue) diameters of the electron‐dense layer. BL, buccal lip; CM, collar membranelles; CSL, concentric sheet layer; CT, curved tubules; EC, electron‐dense core; EL, electron‐dense layer; ER, electron‐dense rings; HT, hemitheca; L, lumen; M, membrane; T‐I, Type I trichites; T‐II, Type II trichites.

Plankton samples were taken with a plankton net (mesh size 10 μm) at the Baltic Sea coast of Warnemünde, Germany (54°10′57″ N, 12°05′15″ E), in October 2017 at a salinity of about 14‰ and a water temperature of about 14°C. *Strombidium biarmatum* (Figure 1A) was identified in vivo by means of light microscopy (Leitz Diaplan microscope), using bright field and interference contrast optics at up to 1250× magnification, and documented by a digital camera (Leica DFC420 camera).

### Preparation for transmission electron microscopy (TEM)

The cells were fixed in a precooled solution of 3% glutaraldehyde in 0.05 M cacodylate buffer (1200 mOsm; pH 7.8) and 2% osmium tetroxide in artificial seawater (30‰) at a ratio of 1:1. After 1 h in ice‐cooled block dishes, the specimens were washed three times with precooled 0.2 M cacodylate buffer for 10 min each. The dehydration was done with precooled ethanol at concentrations of 70%, 80%, 90%, and 96% for 15 min each, followed by three steps with 100% ethanol for 15 min each. Next, the specimens were washed three times for 10 min each in propylene oxide. The embedding process comprised three steps, namely, (i) a propylene oxide to resin (Agar Low Viscosity Resin; Agar Scientific) ratio of 3:1 for 1 h, (ii) a ratio of 1:1 for 2 h, and (iii) a ratio of 1:3 for 5 days. The latter step was conducted in small aluminum dishes that were placed in a desiccator. Polymerization of the samples was performed in an incubator at 40°C for 2 h and finally at 60°C for 24 h. The polymerized samples were ultrathin‐sectioned (70 nm) with an ultramicrotome (Ultracut S, Reichert AG). The investigations were accomplished by means of a Zeiss EM 910 transmission electron microscope (Karl Zeiss AG), and the micrographs were taken with a Sharp:Eye digital camera system (Tröndle), using the computer software ImageSP Viewer (SysProg & TRS). More than 420 pictures from four specimens were taken and analyzed.

### Measurements

Measurements were made by means of the software IC Measure (The Imaging Source). The size of the trichites was measured in organelles that were supposed to show the maximum dimensions. In trichite cross sections, two different diameters of the electron‐dense layer were measured: (i) the diameter of the incircle which tangents to all six outer sides of the hexagon and whose center corresponds to that of the hexagon; (ii) the diameter of the circumcircle which tangents to all six outer edges of the hexagon and whose center again corresponds to that of the hexagon (Figure 1C). Due to the structure's small dimensions, the diameters of the incircle and circumcircle of the electron‐transparent lumen were not distinguishable; thus, the measurement was named diameter despite the minute inaccuracy. In addition, the distance between trichite and its unit membrane as well as the width and thickness of the electron‐dense rings were determined (Figure 1B). The present data base on measurements of three specimens.

### Terminology

The terminology is in accordance with the descriptions of *Strombidium biarmatum* by Agatha et al. (2005) and of the trichite ultrastructure by Modeo et al. (2001). However, the transverse sections of the trichites did not allow the differentiation of the amorphous electron‐dense layer and the concentric sheet layer, and perfect longitudinal sections were insufficient in number. Thus, both layers were generally lumped in the present study into an electron‐dense layer surrounding an electron‐transparent lumen.

### Molecular signature characters

Ganser et al. (2022) suggested the inclusion of molecular signatures as characters in the diagnoses of taxa that are morphologically distinct and provided clear guidelines for their detection and evaluation. We used the tool DeSignate through its web interface (Hütter et al., 2020; https://github.com/DatabaseGroup/DeSignate) to identify potential molecular signature characters which are specified by their position and discrete state (i.e., A, T, G, C, or a gap) in a reference alignment. The consistency of the detected characters can be further evaluated by comparing their presence between the reference and alternative alignments (https://github.com/maxganser/consistency‐script). Here, we used the curated Oligotrichea reference alignment of 18S rRNA gene sequences provided by Ganser et al. (2022) plus the sequence FJ480419 of *Strombidium basimorphum* (Zhang et al., 2010), setting the species *S. biarmatum*, *S. basimorphum*, and *S. paracapitatum* as query group and the remaining oligotrichids as reference group. Further marker gene sequences (e.g., 28S rRNA, ITS regions, or COI) are currently not available for all three species.

The ZooBank registration number of the present work (Recommendation 8A of the International Commission on Zoological Nomenclature, 2012) is urn:lsid:zoobank.org:pub:C3E0F673‐FB05‐4891‐9E6C‐BB6A9B1BA7F6.

## RESULTS

### General morphology of *Strombidium biarmatum*

**FIGURE 2:**
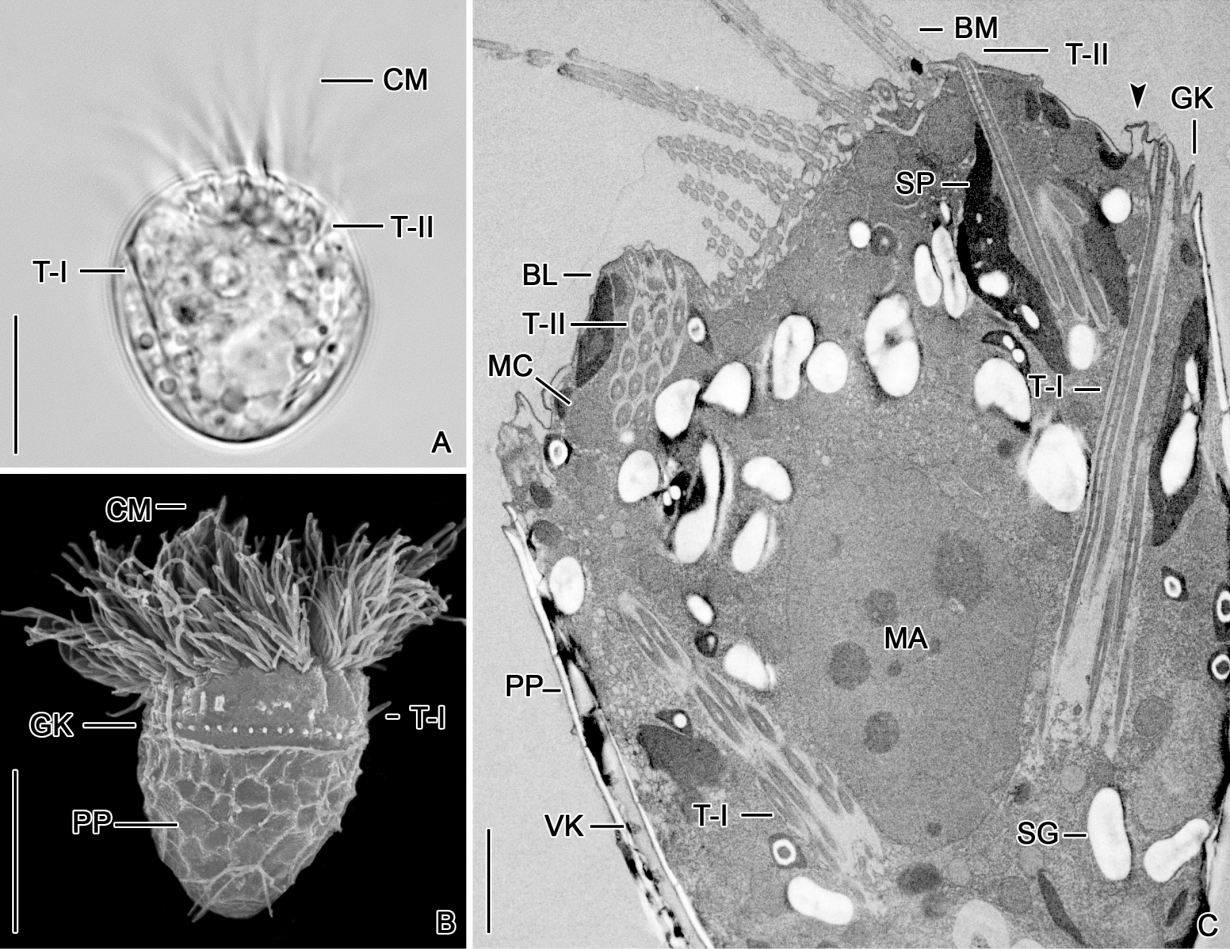
Photomicrographs of *Strombidium biarmatum* from life (A), in the scanning electron microscope (B; from Agatha et al., 2005), and in the transmission electron microscope (C). (A) Lateral view showing the Type I trichites extending obliquely in the cytoplasm of the posterior cell portion and the Type II trichites inserting between the collar membranelles. (B) Lateral view showing just extruding Type I trichites anterior to the girdle kinety and a reticulate pattern generated by the polysaccharide platelets of the hemitheca. (C) Longitudinal section of the anterior cell portion showing cross sections (mainly left side of micrograph) and longitudinal sections (mainly right side) of Type I and Type II trichites. Arrowhead denotes an empty trichite “chamber”. BL, buccal lip; BM, buccal membranelles; CM, collar membranelles; GK, girdle kinety; MA, macronucleus; MC, mitochondria; PP, polysaccharide platelets; SG, starch grains in sequestered plastids or para‐glycogen potentially free in the ciliate's cytoplasm; SP, sequestered plastids; T‐I, Type I trichites; T‐II, Type II trichites; VK, ventral kinety. Scale bars = 10 μm (A, B) and 2.5 μm (C).

**FIGURE 3:**
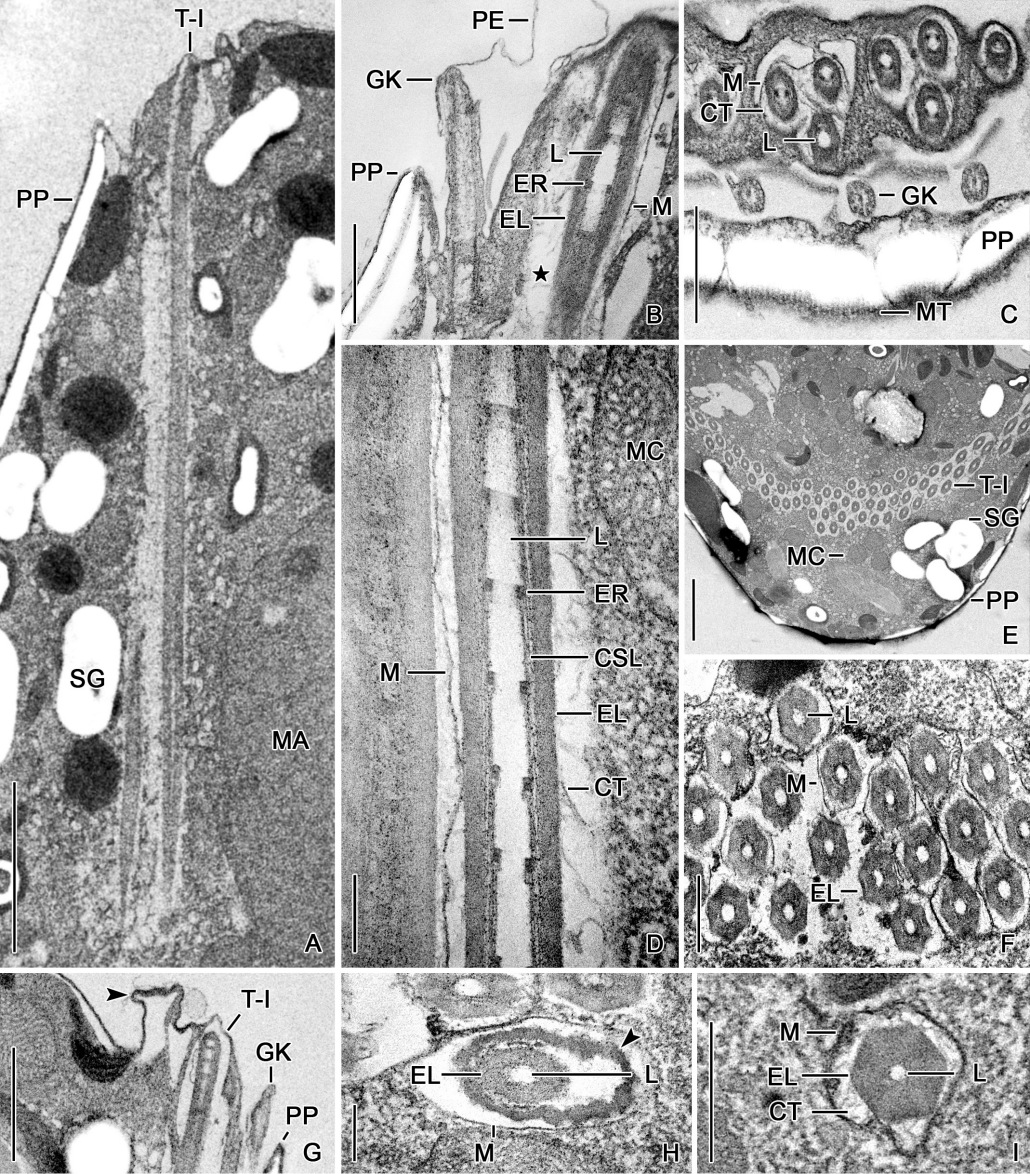
Transmission electron micrographs of Type I trichites in *Strombidium biarmatum*. (A, B, D–G) Longitudinal sections. Asterisk (B) and arrowhead (G) mark empty trichite “chambers”. (C) Tangential cell section shows clusters anterior to girdle kinety. (H, I) Immature trichites with electron‐dense material (arrowhead) apart from (H) or attached to membrane (I). CSL, concentric sheet layer; CT, curved tubules; EL, electron‐dense layer; ER, electron‐dense rings; GK, girdle kinety; L, lumen; M, membrane; MA, macronucleus; MC, mitochondria; MT, microtubules; PE, perilemma; PP, polysaccharide platelets; SG, starch/paraglycogen; T‐I, Type I trichites. Scale bars = 2.5 μm (A, E), 500 nm (B, F, H), 1 μm (C, G), and 250 nm (D, I).

The cell measures about 25 × 20 μm in vivo (*n* = 3) and is broadly obovoidal with an apical protrusion and a rounded posterior portion (Figures 1A and 2A,B). The colorless cytoplasm contains greenish sequestered plastids (kleptoplastids). The C‐shaped adoral zone of membranelles occupies the anterior cell portion, is composed of large collar and small buccal membranelles, and terminates in the buccal cavity (Figures 1A and 2A–C). The membranelles are composed of three rows of basal bodies, except for the proximalmost two or three (buccal) membranelles apparently comprising only two rows (Figures 2C and 5C,G). A buccal lip extends longitudinally on the right side of the buccal field (Figures 1A, 2C, and 5C,D) and bears on its inner side a longitudinally orientated stichomonad endoral membrane (not shown). The dikinetidal girdle kinety extends in a shallow furrow and forms a horizontally orientated circle in the anterior cell half (Figures 2B,C and 3B,C,G); only each left dikinetidal basal body has associated a distinct cilium (not shown). A hemitheca composed of 57–350 nm thick, polygonal polysaccharide platelets is underneath the layer of longitudinally orientated microtubules in the cell portion posterior to the girdle kinety, except for a longitudinal furrow containing the dikinetidal ventral kinety (Figures 1A, 2B,C, 3A–C,E,G, and 5A,F). Only each anterior dikinetidal basal body has associated a distinct cilium in the ventral kinety (not shown). A perilemma encloses the entire cell in addition to the plasma membrane (Figures 3B and 5A,F). The ellipsoidal to broadly ellipsoidal macronucleus is in the posterior portion of the funnel‐shaped complex formed by the Type I trichites, measures about 7.2–11.6 × 5.1–10.8 μm, and contains very few globular nucleoli (Figures 1A, 2C, and 3A). The broadly ovoidal micronucleus is in an indentation of the macronucleus and has a size of about 2.8–3.8 × 1.7–3.5 μm (not shown). The mitochondria are scattered throughout the entire cell with an accumulation in the vicinity of the adoral membranelles. They are broadly ellipsoidal and have tubular cristae (Figures 3D,E and 5B,E,G). Food vacuoles of different sizes contain algal prey in various stages of digestion (not shown). Elongate ellipsoidal sequestered plastids usually extend parallel to the cell surface in the peripheral cytoplasm (Figures 2C and 5E,F). Starch grains are inside the sequestered plastids, while those structures in the cytoplasm might represent paraglycogen (Figures 2C and 3A,E).

### Trichites (oligotrichid extrusomes)

*Strombidium biarmatum* possesses two types of trichites differing in shape, size, and position. The descriptions of these trichites are base exclusively on transmission electron microscopic data.

#### Type I trichites

The arrangement of their attachment sites anterior to the girdle kinety is typical for Oligotrichida, and the extrusive character of this trichite type has been documented in a scanning electron micrograph (Figure 2B). The resting Type I trichites insert in a low, about 1 μm broad circular bulge approximately 1 μm anterior to the girdle kinety (Figures 2C, 3A–C,G, and 5F) and generate with their protruding distal ends a pustulated surface (Figures 2B and 3A,B,G). Electron‐dense material of a spongy texture forms a continuous layer under the cell membrane of the bulge (Figures 3B and 5F). Cytoplasm embeds the distal ends of the individual trichites (Figure 3C); distinct electron‐dense caps are not recognizable. The trichites extend obliquely into the cytoplasm, ending subterminally (Figures 1A, 2C, and 3A,E); thereby, they form a funnel‐shaped complex. Up to four, usually three trichites each form a cluster distally separated by cytoplasm from adjacent clusters in the bulge (Figures 3C and 5E), while the proximal trichite portions are contiguous (Figure 3E,F). Microtubule sheets extending perpendicularly to the bulge surface between the clusters are not visible (Figure 6A).

**FIGURE 4:**
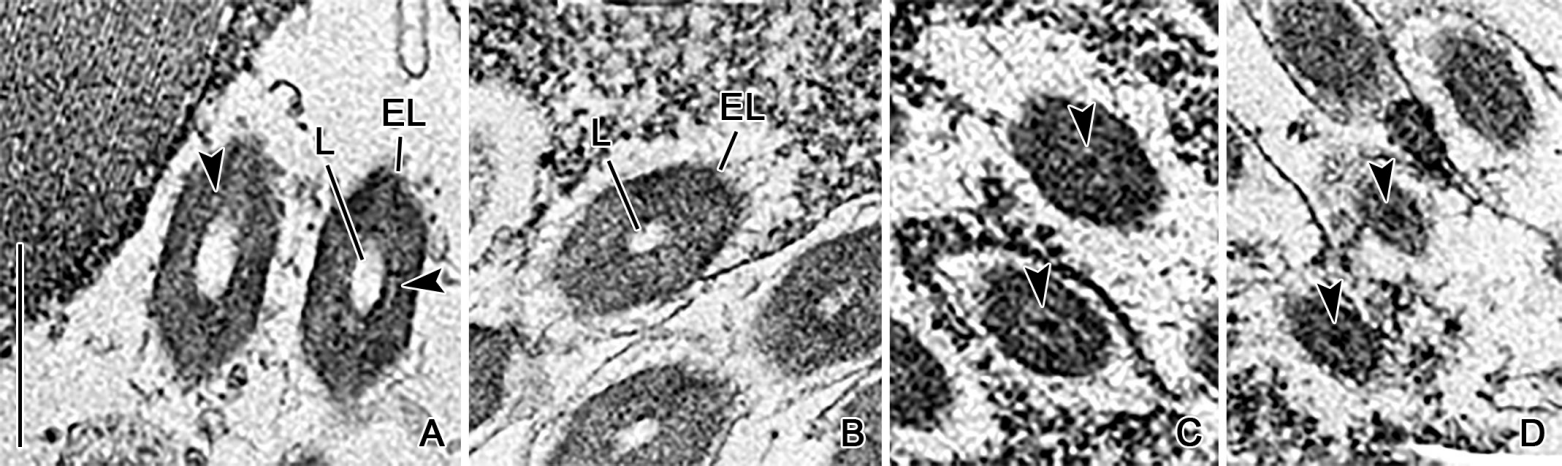
Transmission electron micrographs of *Strombidium biarmatum* showing transverse sections of Type I trichites at different planes from their distal (A) to their proximal (D) ends. Note that the trichites decrease in diameter, and electron‐dense material fills the lumen of the trichites' proximal portions (arrowheads; C, D). Adjacent to the lumen, minute longitudinal lacunas extend (arrowheads; A). EL, electron‐dense layer; L, lumen. Scale bar = 500 nm.

In the resting state, the trichite is on average 12.2 μm long (Figures 2C and 3A; Table 1). Cross sections reveal a hexagonal electron‐dense layer surrounding an indistinctly hexagonal, electron‐transparent lumen, whose proximal portion is filled by an elongate ellipsoidal, electron‐dense core occasionally protruding beyond the trichite's end (Figures 2C, 3C,E,F, 4A–D, and 6A). Infrequently, the edges of the hexagonal electron‐dense layer are slightly more transparent than its sides (Figure 3F,I); distinct sublayers could not be differentiated. The trichite's distal portion has a circumcircle diameter of about 465 nm and a lumen of about 118 nm across (Figures 3B–D,G and 4A; Table 1). The proximal portion is narrowed with a circumcircle diameter of on average 405 nm and a lumen about 107 nm across and has a pointed end; thus, the Type I trichite is acicular (Figures 1B, 2C, 3A, and 4A–D; Table 1). Parallel to the inner sides of the electron‐dense hexagon, minute electron‐transparent longitudinal lacunas extend (Figures 4A,B and 6A).

**TABLE 1:** Morphometric data on the Type I and Type II trichites of *Strombidium biarmatum*.

Character^a^	$\bar{x}$	M	SD	SE	CV	Min	Max	n
Type I trichites (T‐I)								
Length	12.2	12.3	1.33	0.50	10.90	9.3	14.0	7
Distance electron‐dense layer to membrane	50.3	50.2	11.58	4.10	23.05	36.6	68.1	8
Electron‐dense rings, width	22.4	22.0	3.33	0.81	14.86	15.4	28.7	17
Distal portion, diameter of incircle	294.1	277.1	42.09	10.52	14.31	246.5	404.4	16
Distal portion, diameter of circumcircle	465.4	458.7	37.92	9.48	8.15	397.0	553.9	16
Distal portion, diameter of lumen	118.3	114.3	15.60	3.90	13.19	97.5	154.7	16
Proximal portion, diameter of incircle	265.8	259.5	22.87	6.60	8.61	246.7	336.0	12
Proximal portion, diameter of circumcircle	404.6	425.6	35.73	10.31	8.83	335.1	443.0	12
Proximal portion, diameter of lumen	106.5	109.6	15.30	4.42	14.37	66.6	127.6	12
Type II trichites (T‐II)								
Length	6149.5	6178.5	273.51	91.17	4.45	5686.4	6543.1	9
Distance electron‐dense layer to membrane	50.6	50.0	14.30	4.77	28.26	32.8	74.7	9
Electron‐dense rings, width	21.3	21.7	0.65	0.32	3.05	20.2	21.7	4
Diameter of incircle	337.3	330.8	44.48	12.84	13.19	260.1	447.2	12
Diameter of circumcircle	599.4	597.0	107.29	30.97	17.90	421.3	805.6	12
Diameter of lumen	91.2	73.6	33.70	9.73	36.96	56.1	163.4	12

Abbreviations: CV, coefficient of variation in %; *M*, median; Max, maximum; Min, minimum; *n*, number of measurements; SD, standard deviation; SE, standard error of arithmetic mean; $\bar{x}$, arithmetic mean.

^a^ Measurements in nm, except for length of Type I trichites given in μm.

Longitudinal sections occasionally show a thick outer electron‐dense layer and a thin inner concentric sheet layer with telescoped sublayers each merging into a thickened distal margin (Figures 1B and 3D). These margins form electron‐dense rings about 36 nm thick (28–42 nm; *n* = 5), which protrude by about 22 nm into the electron‐transparent lumen (Figures 1B, 2C, and 3B,D,G); the distances between the rings increase from 90 nm in the distal trichite portion to 395 nm in its proximal portion (not shown). A unit membrane encloses the trichite at an average distance of about 50 nm (Figures 1B and 3B,C,D; Table 1). The space between the trichite membrane and the trichite proper is electron‐transparent and contains curved tubules, whose diameter was impossible to measure due to the lack of accurate transverse sections (Figures 1B, 3C,D,F, and 6A).

**FIGURE 5:**
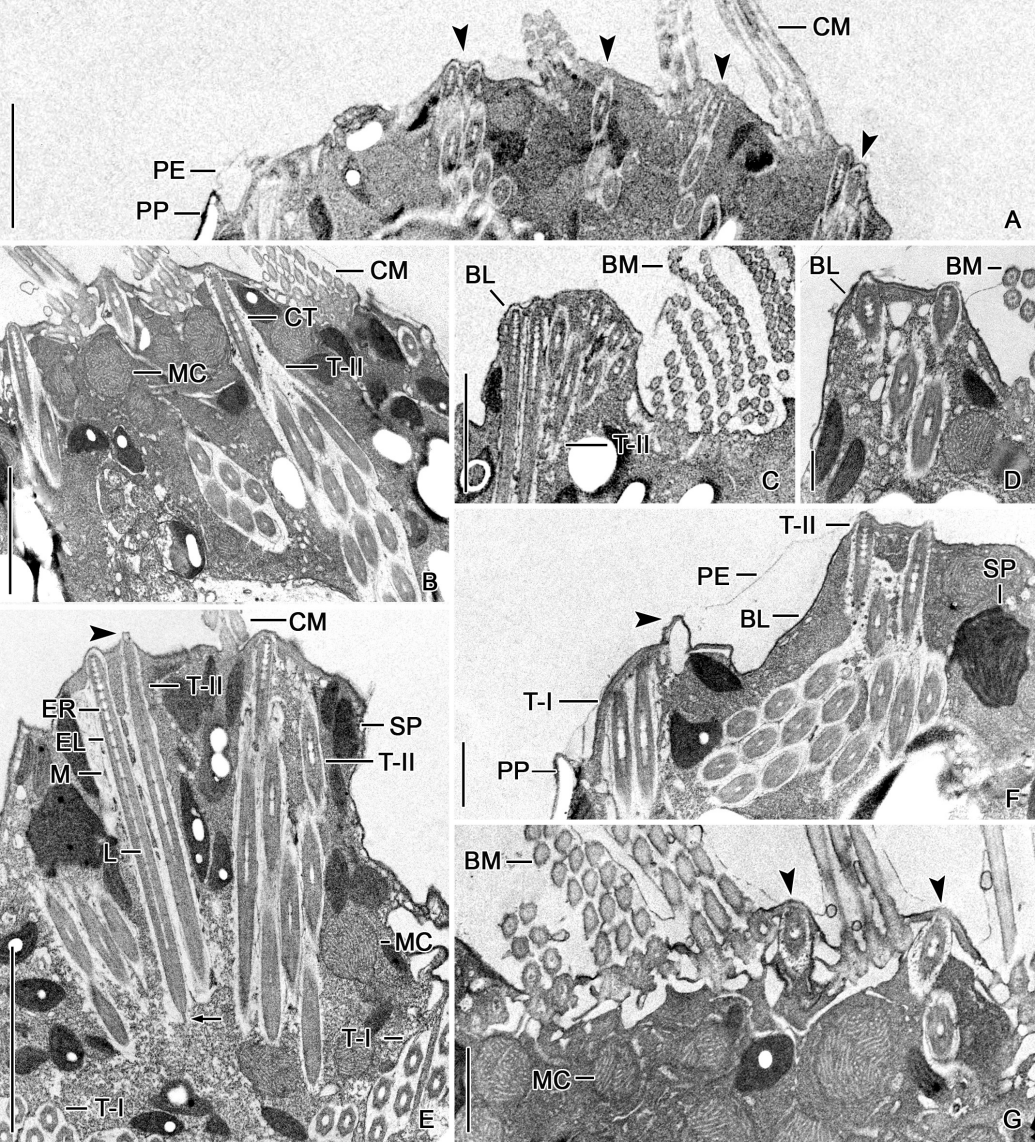
Transmission electron micrographs of *Strombidium biarmatum* showing Type II trichites. (A, B) Details of anterior cell portions. Trichites (arrowheads; A) insert between collar membranelles. (C, D, G) Buccal vertex. Trichites insert in the buccal lip (C, D) and between buccal membranelles (arrowheads; G). (E) Longitudinal section of anterior cell portion. Arrowhead marks an ejecting trichite. An electron‐dense core (arrow) fills the proximalmost portion of the lumen. (F) Cell margin anterior to hemitheca. Arrowhead marks remains of an ejected trichite. BL, buccal lip; BM, buccal membranelles; CM, collar membranelles; CT, curved tubules; EL, electron‐dense layer; ER, electron‐dense rings; L, lumen; M, membrane; MC, mitochondria; PE, perilemma; PP, polysaccharide platelets; SP, sequestered plastids; T‐I, Type I trichites; T‐II, Type II trichites. Scale bars = 2.5 μm (A–C, E), 500 nm (D), and 1 μm (F, G).

Variously shaped protrusions enclosing an optically empty space are visible in some longitudinal cell sections and are interpreted as remains of ejected trichites (Figures 2C, 3G, and 5F). Occasionally, supposedly immature trichites occurred in the posterior cell portion (Figure 3H,I). They already possess an electron‐transparent lumen surrounded by a circular or hexagonal electron‐dense layer whose edges are slightly more transparent than its sides. The trichite proper is enclosed with irregular electron‐dense material up to 80 nm thick, which probably lines the surrounding membrane (Figure 3H,I) but might be artificially separated (Figure 3H). In trichites interpreted as almost mature, curved tubules extend in the space between the electron‐dense enclosure and the hexagonal electron‐dense layer (Figure 3I).

#### Type II trichites

The Type II trichites are short, rod‐shaped, and associated with the adoral zone of membranelles (Figures 1A, 2A,C, and 5A,B,E–G). They overlap with their proximal portions the anterior portions of the Type I trichites (Figures 1A, 2C, and 5E,F). Electron‐dense material of a spongy texture forms a continuous layer under the cell membrane at the attachment sites (Figure 5D–G). Cytoplasm embeds the distal ends of the individual trichites (Figure 5B–G); distinct electron‐dense caps are not recognizable. The distal ends of the trichites protrude, generating a pustulated surface (Figure 5A–G). The trichite clusters inserting in the distal portions of the ridges separating the collar membranelles consist of two rows with two or three, occasionally four organelles each (Figure 5A,B,E). The clusters between the buccal membranelles are less distinct, apparently comprising only a single row with few organelles (Figure 5G); they are absent between the proximalmost membranelles. The cluster in the buccal lip is large, comprising three or four rows of up to five organelles each (Figures 1A, 2C, and 5C,D,F).

In the resting state, the trichite is on average 6.1 μm long (Figure 5E; Table 1). Cross sections reveal a similar structure as in the Type I trichite, i.e., a hexagonal electron‐dense layer surrounds an indistinctly hexagonal, electron‐transparent lumen, which is subterminally filled by an about 1.2 μm long, elongate ellipsoidal, and electron‐dense core occasionally protruding by about 150 nm beyond the trichite's end (Figure 5A–G). Occasionally, the edges of the hexagonal electron‐dense layer are slightly more transparent than its sides (Figure 5F); distinct sublayers could not be differentiated in cross sections. The trichite has a circumcircle diameter of about 599 nm and a lumen of about 91 nm across (Table 1). Thus, they are half the length of Type I trichites, while by on average 29% wider; the lumen diameter is rather similar. Lacunas are not visible.

In longitudinal sections, the electron‐dense layer is separated by an on average 10 nm (6–14 nm; *n* = 5) wide electron‐transparent layer from a concentric sheet layer with telescoped sublayers each merging into a thickened distal margin. These margins form electron‐dense rings about 34 nm thick (21–38 nm; *n* = 4), which protrude by about 21 nm (*n* = 4) into the electron‐transparent lumen (Figures 2C and 5B–F); the distances between the rings increase from about 80 nm in the distal trichite portion to about 300 nm in its proximal portion (Figure 5E). A unit membrane encloses the trichite at an average distance of about 51 nm (Figure 5E; Table 1). The space between the trichite membrane and the trichite proper is electron‐transparent and contains curved tubules 17.5–26.5 nm (*n* = 4) across (Figure 5B). Variously shaped protrusions enclosing an optically empty space are visible in some longitudinal sections and interpreted as remains of ejected trichites.

### Molecular signature characters

No signature characters of the query group comprising *Strombidium biarmatum*, *S. basimorphum*, and *S. paracapitatum* were detected in the Oligotrichea reference alignment of 18S rRNA gene sequences including sequence FJ480419 of *S. basimorphum*. At two “noisy” alignment positions with a low nucleotide variability (average entropy <0.14), only the sequence of *Strombidium capitatum*, which was assigned to the reference group (remaining oligotrichids), shares identical character states (position 1584: A; position 1599: T).

## DISCUSSION

The present ultrastructural data on the trichites in *Strombidium biarmatum* are contextualized in terms of the current state of knowledge about these oligotrichid extrusomes, highlighting those structures that might contribute to a far‐reaching revision of the Oligotrichida beyond the morphological and genetic evidence used here for the establishment of a new genus.

### Comparison with original species description and additional ultrastructural data

*Strombidium biarmatum* had been established based on live observations, protargol‐stained material, scanning electron microscopy, and 18S rRNA gene sequence (AY541684) analyses of specimens collected in the Mediterranean Sea. The two types of trichites differing in their position, length, and shape (Type I in posterior cell half, needle‐shaped, and about 12 μm long vs. Type II associated with adoral membranelles, rod‐shaped, and about 6 μm long) were recognizable only in live cells, while they did not stain by protargol and were rarely visible in overbleached specimens. With the present study, transmission electron microscopic data on the trichites become available showing that the two types otherwise share the same ultrastructure.

The dimensions of the Mediterranean cells in vivo (20–40 × 15–30 μm), their shape, nuclear apparatus, and the presence of sequestered plastids match rather well the data from live observations and optimal longitudinal sections of the Baltic Sea specimens studied here, despite the geographical distance between the sampling sites. While the general arrangement, size, and shape of the two trichite types are congruent between the specimens from the Mediterranean Sea and Baltic Sea (Figure 1A), the present ultrastructural data amend the knowledge about *S. biarmatum*. Agatha et al. (2005) described and depicted a single row of Type I trichites attached to the bulge anterior to the girdle kinety. However, transmission electron microscopy demonstrated a clustering of usually three Type I trichites. Likewise, the Type II trichites are not attached singly or in pairs to the intermembranellar ridges but form groups comprising two rows with up to four organelles each. The Type II cluster in the buccal lip is even larger with up to four rows consisting of up to five organelles each, but had not been observed in the previous study. We attribute these differences to the difficulties of investigating small, highly delicate, and motile cells, but cannot exclude population‐specific deviations.

During live observations, ejected Type I trichites were found to be rod‐shaped and about 60 × 0.5 μm in size. Extruded Type II stages were bipartite in an about 6 μm long, rod‐shaped portion and a filiform portion of similar length; they possibly represent incompletely transformed trichites. Just ejecting trichites were not found in the ultrathin sections; cylindroidal protrusions having attached trichites are interpreted as very early ejection stages, while optically empty protrusions and “chambers” supposedly represent the remains after ejection (Figures 2C, 3G, and 5E,F).

### Ultrastructural comparison of trichites accompanying the girdle kinety

**FIGURE 6:**
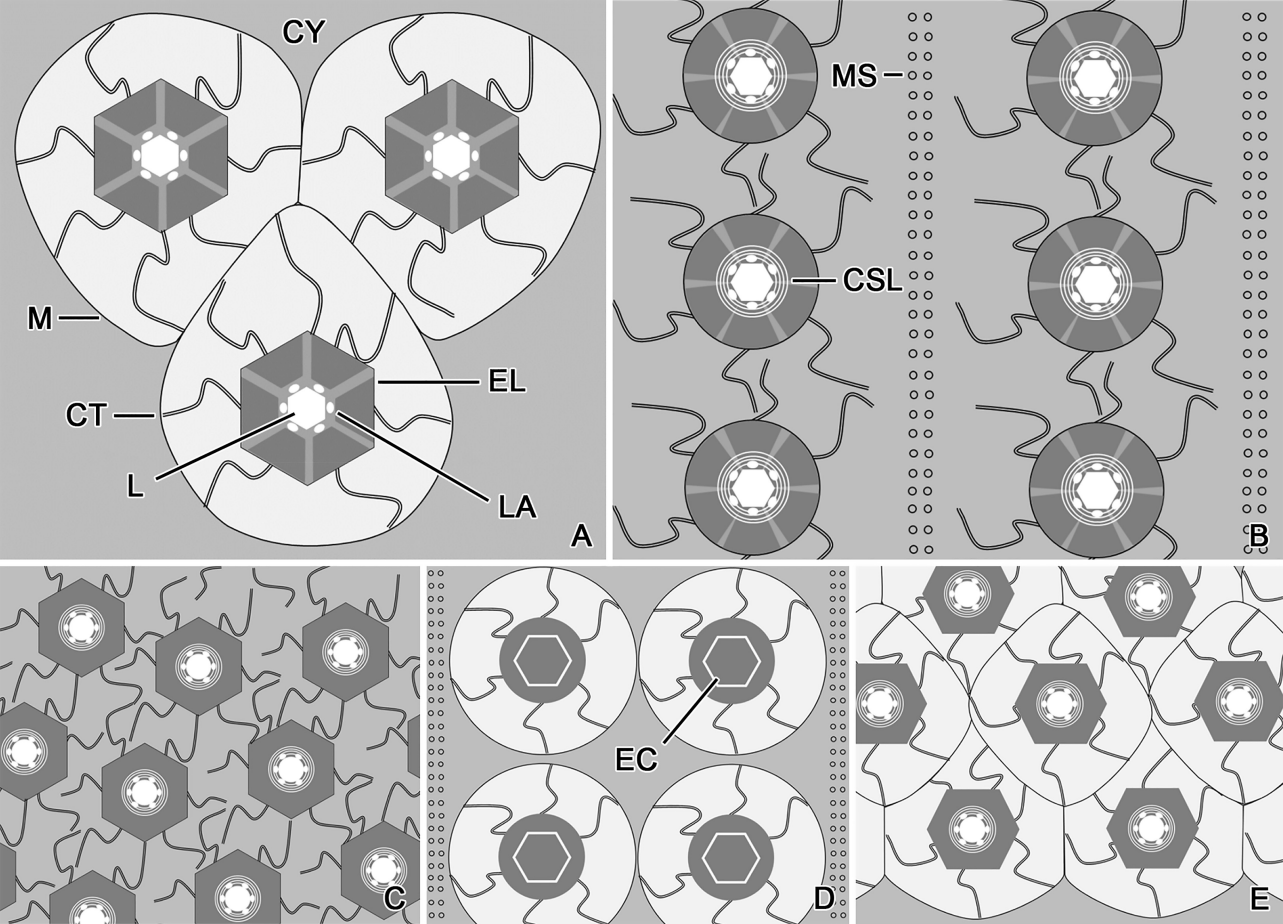
Schemes showing transverse sections of trichites and associated structures accompanying the girdle kinety in strombidiid (A–C), pelagostrombidiid (D), and tontoniid (E) oligotrichids as inferred from literature (B–E). Note that the trichites are not drawn to scale. (A) *Strombidium biarmatum* (this study). (B) *Strombidium inclinatum* and *Novistrombidium testaceum* (Modeo et al., 2001, 2003). The trichite membrane tightly surrounds the trichite proper, and the curved tubules extend in the cytoplasm. The trichite clusters are separated by microtubule sheets. (C) *Strombidium sulcatum* (Fauré‐Fremiet & Ganier, 1970). The trichite membrane tightly surrounds the trichite proper, and the curved tubules extend in the cytoplasm. (D) *Limnostrombidium viride* (Bardele et al., 2018). The curved tubules extend in the space between the trichite membrane and the trichite proper whose lumen is filled by an electron‐dense core. The trichite clusters are separated by microtubule sheets. (E) The secondarily tailless tontoniid *Laboea strobila* (Agatha et al., 2004; Jonsson, 1987). The curved tubules extend in the space between the trichite membrane and the trichite proper. CSL, concentric sheet layer; CT, curved tubules; CY, cytoplasm; EC, electron‐dense core; EL, electron‐dense layer; L, lumen; LA, lacunas; M, membrane; MS, microtubule sheets.

In only a few oligotrichids aside from *Strombidium biarmatum* (this study), trichites have been ultrastructurally studied, namely, in the strombidiids *S. sulcatum* Claparède & Lachmann, 1859, *S. inclinatum* Montag‐nes, Taylor and Lynn, 1990, and *Novistrombidium testaceum* (Anigstein, 1914) Song & Bradbury, 1998, and in the pelagostrombidiid *Limnostrombidium viride* (Stein, 1867) Krainer, 1995; additionally, some anecdotal observations exist for the secondarily tailless tontoniid *Laboea strobila* Lohmann, 1908. In these taxa, trichite attachment sites form a stripe parallel to the anterior margin of the girdle kinety as typical of Oligotrichida. The trichites extend obliquely into the cytoplasm and generate a funnel‐shaped complex in the posterior cell portion of taxa with a horizontal girdle kinety, namely, in *Strombidium* and *Limnostrombidium*. In the following paragraphs, the shared features are shortly summarized, and the main distinguishing features between the Type I trichites of *S. biarmatum* and those of the previously studied species are extracted. Thereby, we demonstrate the potential taxonomic significance of trichite features (Figure 6A–E; Table 2).

**TABLE 2:** Comparison of mature trichites accompanying the girdle kinety in Oligotrichida based on transmission electron microscopic data.

Character	*Strombidiidae*				Pelagostrombidiidae	Tontoniidae
	*Strombidium*			*Novistrombidium*	*Limnostrombidium*	*Laboea*
	*sulcatum*	*inclinatum*	*biarmatum*	*testaceum*	*viride*	*strobila*
Length	15–16 μm	9–12 μm	9.3–14 μm	14–18 μm	17 μm	17–24 μm^a^
Diameter circumcircle	378 nm	350 nm	397–554 nm	450 nm	500 nm	970 nm
Diameter incircle	360 nm	?	247–404 nm	?	?	810–870 nm
Shape	Rod‐shaped	Rod‐shaped	Needle‐shaped	Rod‐shaped	Needle‐shaped	Needle‐shaped
Arrangement	?	Single or paired rows of 6–8 T each directly anterior to GK	Clusters of usually 3 T each directly anterior to GK	14 clusters of 8–10 T each parallel to but distinctly apart from GK	12–16 clusters of 5 or 6 paired rows of 3 or 4 T each directly anterior to GK	Single or paired rows of up to 7 T each directly anterior to GK
Electron‐dense material underneath cell membrane	+	+	+	+	+	+
Cytoplasm separating distal portions	+	+	+	+	+	?
Electron‐dense layer, width	?	80 nm	~ 85–175 nm^*^	30 nm	110 nm^*^	130 nm^*^
Concentric sheet layer, width	?	40 nm		130 nm		
Lumen, diameter	108 nm	110 nm	98–155 nm, core in proximal portion	150 nm	~ 190 nm, core in entire organelle	290–355 nm
Electron‐dense rings, width	+	20 nm	22 nm	−	−	?
Electron‐dense rings, maximum distance	370 nm	?	395 nm	n.a.	n.a.	?
Position of curved tubules in relation to membrane	outside	outside	inside	outside	inside	inside
Cytoplasmic pockets	−	−	+	−	+	+
Microtubule sheets separating clusters	No evidence	+	No evidence	+	+	?
Reference	[1]	[2, 4]	[3]	[2, 4]	[5]	[6, 7]

Abbreviations: GK, girdle kinety; n.a., not applicable; T, trichites; VK, ventral kinety; +, present, but no details provided; −, absent;?, unknown state. [1] Fauré‐Fremiet and Ganier (1970); [2] Modeo et al. (2001); [3] present study; [4] Modeo et al. (2003); [5] Bardele et al. (2018); [6] Agatha et al. (2004); [7] Jonsson (1987).

^a^ In vivo.

^*^ Total width of both electron‐dense and concentric sheet layers.

All the trichites accompanying the girdle kinety studied yet fall into the same size range (9–24 μm long, 350–700 nm wide), have an elongated shape (rod‐shaped or needle‐shaped), are membrane‐bound, and have associated curved tubules. Usually, cross sections show an electron‐transparent lumen surrounded by an inner concentric sheet layer and an outer electron‐dense hexagon (*S. biarmatum*, *S. sulcatum*, *Limnostrombidium viride*, *Laboea strobila*) or ring (*S. inclinatum*, *N. testaceum*). Small electron‐transparent lacunas extend longitudinally and parallel to the inner sides of the hexagon in some (possibly all) species. The cell membrane is underlaid by electron‐dense material of a spongy texture in the stripe of attachment sites.

*Strombidium inclinatum* (Figure 6B; Table 2; reported as S3 in Modeo et al., 2001, 2003). The trichites are indistinctly clustered. Electron‐dense caps cover the trichites' distal ends, while the remaining cytoplasm is fluffy. Double sheets of microtubules extend parallel to the trichites' distal fifths, separating the clusters. The curved tubules extend in the cytoplasm, i.e., outside the membrane tightly surrounding the trichite.

*Strombidium sulcatum* (Figure 6C; Table 2; Fauré‐Fremiet & Ganier, 1970). The first transmission electron microscopical study on an oligotrichid ciliate was conducted in the type species of the genus *Strombidium*. Its trichites insert exclusively anteriorly to the horizontal girdle kinety; details are not provided. Electron‐dense caps cover the trichites' distal ends, while the remaining cytoplasm is fluffy. The curved tubules extend in the cytoplasm, i.e., outside the membrane tightly surrounding the trichite. Microtubule sheets are neither mentioned in the text nor shown in the micrographs.

*Novistrombidium testaceum* (Figure 6B; Table 2; reported as S1 in Modeo et al., 2001, 2003). In this strombidiid genus, the trichite attachment sites are parallel to but distinctly apart from the girdle kinety, i.e., they form a dextrally spiraled pattern near the posterior ends of the adoral membranelles (Modeo et al., 2003; Song & Bradbury, 1998). The trichites of this stripe are distinctly clustered; three or four additional clusters are associated with the longitudinal ventral kinety (Modeo et al., 2003). Electron‐dense caps cover the trichites' distal ends, while the remaining cytoplasm is fluffy. Double sheets of microtubules extend parallel to the trichites' distal fifths, separating the clusters. The curved tubules extend in the cytoplasm, i.e., outside the membrane tightly surrounding the trichite. Electron‐dense rings are not present. In the congener *N. apsheronicum*, the trichite pattern is similar, except for the additional trichites which form a subterminal arc (Agatha, 2003).

*Limnostrombidium viride* (Figure 6D; Table 2; Bardele et al., 2018). This species is the only representative of the oligotrichid family Pelagostrombidiidae whose trichites were ultrastructurally investigated. The trichites are hierarchically grouped, i.e., five or six paired rows form a cluster; such an arrangement has not been described in the other ultrastructurally studied oligotrichids. The double rows of a cluster are separated by cytoplasm containing two or three sheets of parallel microtubules, which might originate in the margins of the distinct electron‐dense caps covering the trichites' distal ends. Interestingly, the lumen of mature trichites is filled by an electron‐dense hexagonal core about 190 nm across, resembling the core in the proximal portion of *S. biarmatum* trichites. In contrast, supposedly immature trichites have an electron‐transparent lumen. Lacunas and electron‐dense rings are not visible in the mature trichites. The curved tubules extend in the space between the trichite membrane and the trichite proper. In the confamilial, species *Pelagostrombidium mirabile* (identified as *L. viride* although it lacks somatic cilia and has a long buccal portion of adoral membranelles), the trichites were misinterpreted as nematodesmata (Rogerson et al., 1989). These trichites also possess electron‐dense cores which thus potentially characterize the family Pelagostrombidiidae.

*Laboea strobila* (Figure 6E; Table 2; Agatha et al., 2004; Jonsson, 1987). Anecdotal observations are available for the monotypic, secondarily tailless tontoniid genus *Laboea* with its sinistrally spiraled girdle kinety. The gradual decrease in trichite length from about 24 μm in the anterior whorls to about 17 μm in the posterior whorls was estimated by live observations (Agatha et al., 2004), while the ultrastructural features have been measured by us in the transmission electron micrograph (Figure 4h) published by Agatha et al. (2004). Scanning electron micrographs show an indistinct clustering of the trichite attachment sites, forming single‐ or two‐rowed units with up to seven organelles each. However, the transmission electron micrograph displays a more or less contiguous trichite arrangement with only very thin extensions of cytoplasm separating the distal portions of the single or double rows. Whether the separating cytoplasm contains microtubule sheets is not visible in the micrograph. Probably, electron‐dense caps cover the trichites' distal ends. Since longitudinal sections are not available, the presence of electron‐dense rings is uncertain.

The main ultrastructural differences of the trichites in the abovementioned taxa (Table 2) concern (i) the position of the curved tubules with respect to the trichite membrane (inside vs. outside), (ii) the microtubule sheets (present vs. absent), (iii) the electron‐dense core filling the lumen of mature trichites (present in entire lumen vs. restricted to proximalmost portion vs. absent), (iv) the electron‐dense rings (present vs. absent), and (v) the clustering (absent vs. simple vs. hierarchical). Probably, these are taxonomically relevant features that might contribute to a more far‐reaching revision of the Oligotrichida, whereas the trichites' size is considered species‐specific. Preparation methods might influence the recognizability of lacunas and concentric sheet layers, for instance, in transverse sections of trichites; thus, such deviations should not be overestimated at the current state of knowledge.

Light microscopical studies on several live and preserved oligotrichids revealed “trichite pockets” (e.g., “Taschen mit Trichocysten” sensu Kahl, 1932; “plasma pockets” sensu Krainer, 1991). The impression of pockets might result from contiguous trichites having a space between the organelles and their enclosing membranes as in *S. biarmatum* (Figure 6A), *Limnostrombidium viride* (Figure 6D), and *Laboea strobila* (Figure 6E). Further data are required for reconciling the structures revealed by light and transmission electron microscopy, e.g., the nature of the inverted L‐shaped, argyrophilic lines associated with the trichite attachment sites in protargol stains of several oligotrichids (e.g., *Laboea strobila*; Agatha et al., 2004).

### Comparison of taxa with additional trichites

Since protargol‐staining reveals trichites only insufficiently and might cause their deformation (e.g., they might become tear‐shaped in *Laboea strobila*, *Omegastrombidium elegans*, *Parallelostrombidium siculum*, *Pseudotontonia simplicidens*, *Strombidium acutum*; Agatha et al., 2004; Liu et al., 2011; Montagnes & Taylor, 1994; Song et al., 2000), live observations are indispensable for the investigation of the organelles' size and arrangement. Thorough studies also necessitate the application of transmission electron microscopy. Here, we provide the first ultrastructural data on trichites associated with adoral membranelles. Hence, the comparison of our findings in *Strombidium biarmatum* with those from the three congeners possessing additional trichites in the anterior cell portion (*S. basimorphum*, *S. paracapitatum*, *S. rehwaldi*) is restricted to live observations.

*Strombidium rehwaldi* had been described from freshwater (Petz & Foissner, 1992). Its additional trichites are indistinctly clustered and their attachment sites form a stripe directly posterior to the membranelles. They are identical in size and shape to the ones of the funnel‐shaped complex in the posterior cell portion. Gene sequence data are missing.

*Strombidium paracapitatum* and *S. basimorphum* share additional trichites between the adoral membranelles with *S. biarmatum*. The three species cluster with high/maximum statistical support in phylogenetic trees based on 18S rRNA gene sequences (Li et al., 2020; Santoferrara et al., 2017; Song et al., 2015, 2019, 2021) indicating their close relationship.

Live observations on *S. paracapitatum* revealed the trichites of the funnel‐shaped complex to be longer than the additional ones (about 18 μm vs. about 10 μm) which insert singly or in pairs in the distal portions of the intermembranellar ridges (Song et al., 2015). In protargol‐stained cells, they are apparently not visible.

*Strombidium basimorphum* had been described based on protargol‐stained material collected from a British Columbian fjord (Martin & Montagnes, 1993). The authors noticed faintly stained trichites forming the typical funnel‐shaped complex in the posterior cell portion; further trichites were not mentioned, and genetic data were not available. Based on a similar cell morphology, Liu et al. (2011) redescribed the species from Chinese coastal waters with additional trichite clusters between the adoral membranelles recognizable in live specimens. While the authors were unable to detect such structures in the type material at that time (Liu et al., 2011), the recent reinvestigation of their micrographs displaying the protargol‐stained Canadian specimens confirmed the presence of trichites inserting between the adoral membranelles (Weiwei Liu, pers. commun.). Despite the huge geographic distance between the fjord on the Canadian west coast and the mangrove wetland on the Chinese east coast (about 10,000 km), the affiliation of the sampling sites to climatically different Longhurst provinces (California Current Province vs. China Sea Province), the different water temperatures (6–9°C vs. 27°C), and the deviating number of buccal membranelles (5–7 vs. 7–9), the specimens described by Martin and Montagnes (1993) and Liu et al. (2011) are thus supposedly conspecific. *Strombidium basimorphum* possesses two types of rod‐shaped trichites differing in length (Type I about 20 × 0.8 μm, Type II about 15 × 0.8 μm; Liu et al., 2011). Since it is important to link gene sequence data with species descriptions and sampling sites, we revised the literature data. The genetic material analyzed by Li et al. (2013; ITS1, 5.8S rRNA, and ITS2; accession number JN853787) and Zhang et al. (2010; 18S rRNA gene sequence with correct accession number FJ480419) was extracted from cells belonging to the same population as the morphologically studied Chinese specimens (Weiwei Liu, pers. commun.). The coordinates of the Chinese sampling sites given in the previously mentioned publications erroneously deviate; the correct sampling site for the whole material is 22°32′ N, 114°01′ E (Weiwei Liu, pers. commun.).

The occurrence of additional trichites is not restricted to the anterior cell portion. In some oligotrichid species, they insert along the ventral kinety (*Novistrombidium testaceum*, *Strombidium lingulum*; Modeo et al., 2003; Montagnes & Humphrey, 1998) or in an arc in the posterior cell portion and separate from the somatic ciliature (*N. apsheronicum*; Agatha, 2003).

### Formation of trichites

To our knowledge, there are no studies about the development of trichites. Ribosomes in the organelles' vicinity indicate intense protein production (Fauré‐Fremiet & Ganier, 1970; Modeo et al., 2001). Rosati and Modeo (2003) suggested an involvement of the endoplasmic reticulum or the Golgi complex in their formation. Currently, we can infer the trichite formation merely from anecdotal observations of their immature stages.

In the pelagostrombidiid *Limnostrombidium viride*, the immature trichite is enclosed by a membrane and situated in the indentation of an electron‐dense, reniform structure (Bardele et al., 2018). At this stage, they look like a flower with six concentrically striped petals arranged around an electron‐transparent lumen in transverse sections; the electron‐dense rings are indistinct. The immature trichites of the tontoniid *Laboea strobila* are also situated in an indentation of an electron‐dense structure (Agatha et al., 2004). In contrast, the immature trichites of *S. biarmatum* are not placed in the indentation of an electron‐dense structure but are seemingly enclosed by thick, electron‐dense material (Figure 3H,I). In an immature state, the pelagostrombidiid trichites resemble mature trichites in the strombidiids *S. sulcatum*, *S. inclinatum*, and *S. biarmatum*, and the tontoniid *Laboea strobila* regarding the electron‐transparent lumen. Accordingly, not only the trichite ultrastructure but also its genesis might provide further taxonomic features.

### Trichite ejection and ultrastructural changes

Direct and indirect evidence for the ejection of trichites is given by their observed extrusion (see “Introduction”) and the “8 + 1”‐attachment rosettes in *Limnostrombidium viride* typical for extrusive ciliate organelles (Bardele et al., 2018), respectively. Our observations of telescoped concentric layers fit the findings of Modeo et al. (2001) whose micrographs indicate a trichite elongation (5×) during ejection by a sliding of the concentric sheet layer out of the electron‐dense layer. Likewise, the ejected trichites are elongated and filiform in *Strombidium biarmatum* (5×) and *Laboea strobila* (16×; Agatha et al., 2004). The optically empty protrusions and “chambers” in the cells are congruently interpreted as remains after an ejection (this study; Bardele et al., 2018; Modeo et al., 2001; Rosati & Modeo, 2003). The anecdotal observations of ejected Type II trichites in *S. biarmatum* possibly base on incompletely exploded organelles with a filiform anterior and a rod‐shaped posterior portion.

### Chemical composition

The nature of trichites has been debated for some time. Gourret and Roeser (1888) suggested a chitinous material, whereas Fauré‐Fremiet and Ganier (1970) proposed a proteinaceous nature. Finally, Modeo et al. (2001) detected mainly proteinaceous material in the trichites of the strombidiid *S. inclinatum*. The application of protease and pepsin each caused a partial digestion, indicating differences in the chemical composition of the electron‐dense layer, the cap material, and the electron‐dense rings. Even the six more electron‐transparent zones extending radially from the lumen to the trichite membrane and the hexagon's sides as well as the concentric sheet layer are chemically different. A polysaccharide‐specific stain revealed the electron‐dense rings, the trichite membrane, and the curved tubules (Modeo et al., 2001).

### Taxonomic and nomenclatural acts

The type species of the genus *Strombidium*, *S. sulcatum*, had originally been described from the Bergen Fjord on the Norwegian North Sea coast by Claparède and Lachmann (1859). Since the original description does not allow an unequivocal identification, the first detailed redescription of specimens from the French North Atlantic coast by Fauré‐Fremiet (1912) has been considered authoritative by Granda and Montagnes (2003). These authors deposited a neotype from the material collected and stained by Fauré‐Fremiet. The specimens ultrastructurally studied by Fauré‐Fremiet and Ganier (1970) were sampled at various sites, including the French North Atlantic coast, and matched the previous findings. These detailed light and transmission electron microscopic studies congruently detected a funnel‐shaped complex of trichites in the posterior cell portion but no further trichites. The available gene sequences (accession numbers DQ777745, FJ377546; Yi and Song, unpubl.; Zhang et al., 2010) are from Chinese coastal waters distinctly apart from the French type locality. The conspecificity of these specimens cannot be confirmed based on the illustrations given and is questionable considering the small percentage of amplicons shared between European and Chinese coastal waters (Ganser et al., 2021).

The establishment of new ciliate taxa should preferably be based on several lines of evidence, for instance, morphologic and genetic distinctness. In contrast to the type species *Strombidium sulcatum*, the species *S. basimorphum*, S. *biarmatum*, and *S. paracapitatum* are characterized by a strong morphologic synapomorphy, viz., trichites not only inserting anteriorly to the girdle kinety but also between the adoral membranelles. This feature is corroborated by the consistent formation of a statistically well‐supported monophylum in 18S rRNA gene sequence analyses (Li et al., 2020; Santoferrara et al., 2017; Song et al., 2015, 2019, 2021). Based on these morphologic and genetic data, the new genus *Heteropilum* nov. gen. is proposed for *S. biarmatum*, *S. basimorphum*, and *S. paracapitatum*. The lack of molecular signature characters in the query group comprising *Heteropilum biarmatum* nov. gen., nov. comb., *H. basimorphum* nov. comb., and *H. paracapitatum* nov. comb. might be the result of plesiomorphies and/or multiple substitutions at two alignment positions (position 1584: A and position 1599: T) in a single sequence assigned to the reference group, namely, in *Strombidium capitatum*. In the future, further currently not available marker genes (e.g., the D1/D2 regions of the 28S rRNA gene) might provide molecular signature characters for complementing the taxonomic diagnosis of the genus introduced below based on the unique possession of additional trichites inserting between the adoral membranelles.

## TAXONOMIC SUMMARY

Class Oligotrichea Bütschli, 1887.

Order Oligotrichida Bütschli, 1887.

Family Strombidiidae Fauré‐Fremiet, 1970.

***Heteropilum* nov. gen.**

**Diagnosis.** Strombidiidae with additional trichites inserting between the adoral membranelles.

**Type species.** *Strombidium biarmatum*.

**Etymology.** Composite of the prefix *hetero*‐ and the Latin noun *pilum* (spear) referring to the two types of trichites; neuter gender.

**Assigned species.** Besides the type species *Heteropilum biarmatum* nov. gen., nov. comb., the following species are affiliated: *Heteropilum basimorphum* nov. comb. (basionym: *Strombidium basimorphum* Martin & Montagnes, 1993) and *Heteropilum paracapitatum* nov. comb. (basionym: *Strombidium paracapitatum* Song et al., 2015). As for congenerity of the freshwater species *Strombidium rehwaldi* Petz & Foissner, 1992 with its stripe of additional trichite attachment sites directly posterior to the adoral membranelles, further genetic and ultrastructural investigations are needed.

**ZooBank registration number.** urn:lsid:zoobank.org:act:D4D2E8F2‐9E33‐45CF‐8D7B‐8350B1B29A67.
